# Tear IgE point-of-care testing for differentiating type I and type IV allergic conjunctivitis

**DOI:** 10.3389/fmed.2025.1577656

**Published:** 2025-05-26

**Authors:** Xumin Shang, Yiqiu Zhang, Shunrong Luo, Miaomiao Liu, Hanqiao Li, Xie Fang, Zhiwen Xie, Xianwen Xiao, Zhengwei Yang, Yuan Lin, Huping Wu

**Affiliations:** ^1^Xiamen Eye Center and Eye Institute of Xiamen University, School of Medicine, Xiamen, China; ^2^Xiamen Clinical Research Center for Eye Diseases, Xiamen, Fujian, China; ^3^Xiamen Key Laboratory of Ophthalmology, Xiamen, Fujian, China; ^4^Fujian Key Laboratory of Corneal & Ocular Surface Diseases, Xiamen, Fujian, China; ^5^Xiamen Key Laboratory of Corneal & Ocular Surface Diseases, Xiamen, Fujian, China; ^6^Translational Medicine Institute of Xiamen Eye Center of Xiamen University, Xiamen, Fujian, China

**Keywords:** IgE, POCT, tear, allergic conjunctivitis, conjunctivitis scores

## Abstract

**Objective:**

This study aimed to evaluate the diagnostic utility of point-of-care tear immunoglobulin E (IgE) testing in distinguishing between type I and type IV allergic conjunctivitis (AC), and to explore tailored treatment strategies.

**Methods:**

A total of 254 patients with perennial AC at Xiamen Eye Center were enrolled. Clinical data, including age, sex, symptoms, and signs, were collected. Tear total IgE was measured using the i-ImmunDx™ platform. Univariate and multivariate logistic regression analyses, along with ROC curve analysis, were used to assess the discriminative value of tear IgE and clinical parameters for AC subtypes.

**Results:**

The mean patient age was 14.11 ± 12.46 years; 182 were male. Mean conjunctivitis score was 4.09 ± 1.51, and mean tear IgE was 7.73 ± 16.76 IU/mL. Tear IgE levels negatively correlated with age (*p* < 0.05), and positively with conjunctival secretion, papillary hyperplasia, and conjunctivitis scores (*p* < 0.05). Univariate analysis showed age, tear IgE, and papillae were significantly associated with type IV AC. Multivariate analysis identified tear IgE, conjunctival papillae, and conjunctivitis score as independent predictors. ROC analysis showed an AUC of 0.896 for tear IgE (cut-off = 5.57 IU/mL; sensitivity 89.00%, specificity 77.78%). A combined model (IgE + papillae + score) improved AUC to 0.912, with sensitivity of 81.50% and specificity of 88.89%.

**Conclusion:**

Tear IgE effectively differentiates AC subtypes and correlates with disease severity. Patients with low IgE levels, indicative of type IV hypersensitivity, benefit from individualized anti-inflammatory therapies, supporting its role in personalized management.

## Introduction

Allergic conjunctivitis (AC) is a prevalent ocular condition, characterized by itching, foreign body sensation, light sensitivity, and excessive tearing. It primarily results from hypersensitivity reactions to allergens, which trigger the release of inflammatory mediators (1). Damage to the epithelial barrier facilitates allergen absorption, leading to immune activation and the development of chronic inflammation, a common feature of allergic diseases (2). Humid environments significantly increase the risk of AC, particularly in persistent humid conditions (3). In tears, pro-inflammatory cytokines are elevated in perennial allergy patients, while MMP-9 and IgA levels increase in those with seasonal allergies (4). Currently, studying tear components in the local immune-inflammatory response of AC offers promising research directions for clinical treatment and evaluation (5).

AC is primarily caused by The immunoglobulin E (IgE)-mediated type I hypersensitivity reactions (6). Upon re-exposure to an allergen, FcεRI receptors on mast cells, eosinophils bind and cross-link with the allergen, triggering mast cell degranulation (7). The process releases various inflammatory mediators, including histamine, leading to immediate allergic symptoms such as ocular itching and a foreign body sensation, often accompanied by white mucous discharge from the conjunctival sac (8). Clinically, conjunctival hyperemia is the most frequent sign, often along with varying degrees of conjunctival edema (9). The central role of IgE in AC is underscored by its ability to trigger allergic reactions in the eyes. While the severity of allergic responses is typically assessed through serum IgE levels, measuring total IgE levels in tears may offer a more direct evaluation of ocular allergic reactions (10–12). AC patients have higher tear IgE concentrations than healthy individuals, with a significant correlation between tear and serum IgE levels. Elevated tear IgE levels in seasonal (SAC), perennial (PAC), and vernal keratoconjunctivitis (VKC) indicate allergic conjunctival reactions, while levels in epidemic keratoconjunctivitis (EKC) and bacterial conjunctivitis (BC) are similar to those in healthy controls (13). In clinical practice, type IV AC is often misdiagnosed as seasonal type I AC. This is because seasonal allergic conjunctivitis (SAC) typically occurs during specific seasons or after exposure to specific allergens, whereas type IV AC can persist and is associated with continuous exposure to allergens. As a result, the persistent symptoms of type IV AC may be mistaken for SAC, especially at the initial diagnosis.

At present, point of care testing (POCT) technology is widely used in clinical practice, which is helpful for clinical workers to evaluate and develop personalized treatment plans, so as to achieve the purpose of precision medicine (14). POCT technologies are used to obtain molecular biological information rapidly for the screening and differential diagnosis of conditions like dry eye, adenoviral conjunctivitis, and endophthalmitis. These tests detect specific biomarkers or pathogens, allowing clinicians to quickly distinguish between similar ocular conditions. For instance, in dry eye, inflammatory markers can be assessed, while adenoviral conjunctivitis can be identified by viral DNA/RNA, aiding in early diagnosis and targeted treatment decisions (15–17). Through tear POCT technology, molecular-level immune inflammation information can be quickly obtained in clinical practice (18). This study evaluates the diagnostic value of tear total IgE levels based on the i-ImmunDx™ platform which incorporates a micro-capillary fluid collector, POCT diagnostics, and an advanced analyzer.

## Methods

### Study design and population

**Figure 1:**
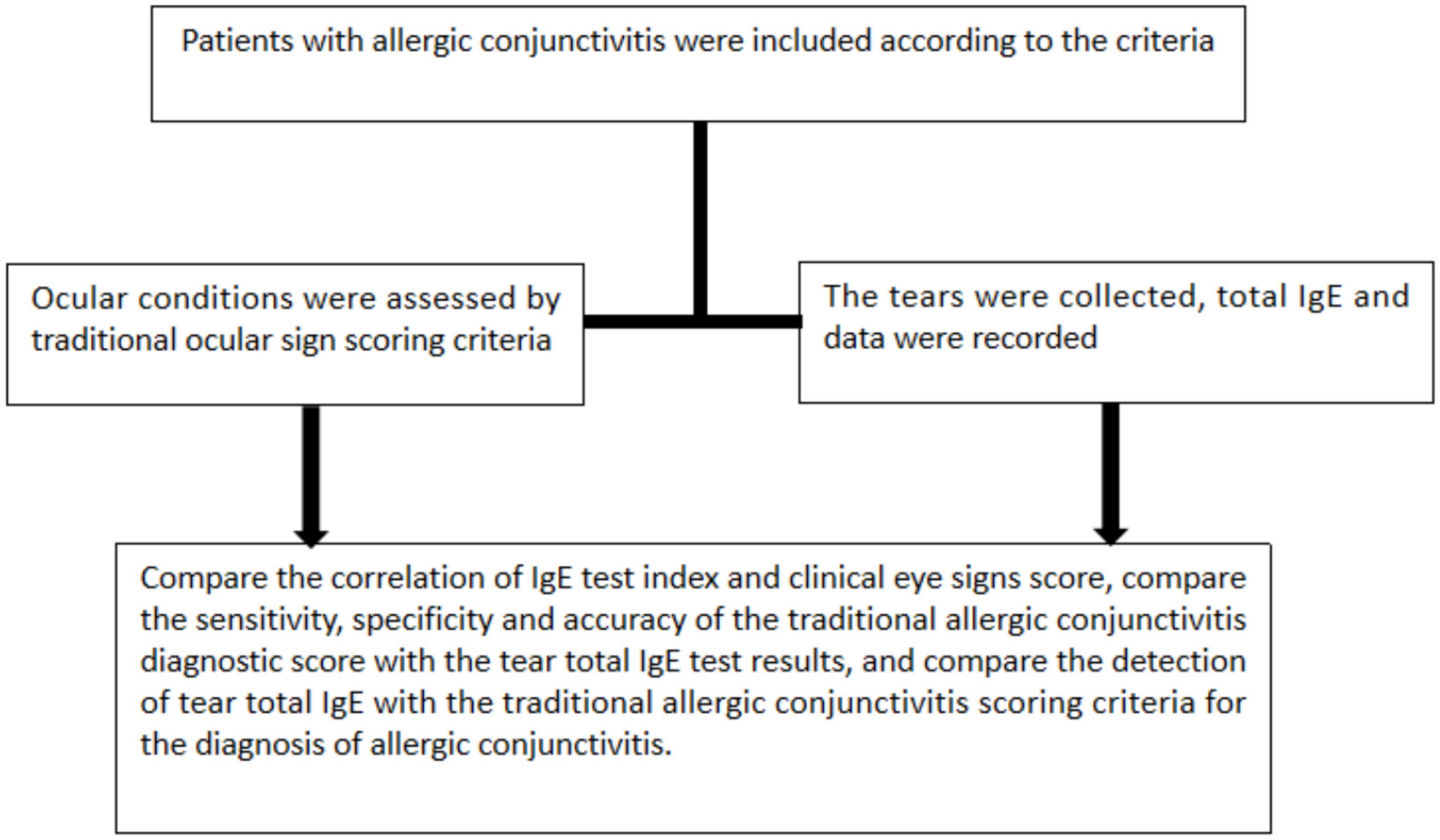
The technical roadmap of this study. After screening patients by inclusion and exclusion criteria, non-IgE and IgE AC patients were analyzed for collecting clinical symptoms and scoring of AC patients and collecting total tear IgE using POCT.

This retrospective study was approved by the Human Ethics Committee of Xiamen Ophthalmology Center. Informed consent was waived due to the retrospective nature of the study. A total of 254 patients diagnosed with AC were included and separate groups based on tear IgE results. Patient demographics, ocular symptoms, and signs were collected. All patients underwent rapid tear IgE testing (Figure 1). The tear IgE test results were systematically recorded in the testing device or electronic medical records, making them retrievable for retrospective analysis. Rhinoconjunctivitis Quality of Life Questionnaires (RQLQs) were used during the initial outpatient visit to assess the patients’ allergy-related conditions (19). All evaluations in this study were performed by a single ocular surface specialist (Xumin Shang).

### Inclusion and exclusion criteria

Inclusion criteria for the study required participants to meet the one of following conditions: a documented history of allergic symptoms within 24 months prior to screening a confirmed positive result from at least one diagnostic test including skin prick test intradermal test or serum-specific IgE antibody test detection of eosinophils in conjunctival scrapings. A clinical diagnosis of AC characterized by itching, foreign body sensation, serous or mucous discharge, and conjunctival hyperemia (20) includes an eye itching score of 3 or higher and a conjunctival hyperemia score of 1 or higher and willingness to adhere to the study’s examination and treatment protocols.

Exclusion criteria included any ocular diseases that could interfere with AC evaluation, such as trauma, keratitis, ophthalmitis, conjunctivitis, dry eye, glaucoma, or corneal and retinal disorders. Active infections (viral, bacterial, fungal, or protozoal), a history of ocular herpes, or the need for unrelated eye treatments were excluded. Patients who had used corticosteroids, antihistamines, NSAIDs, or leukotriene receptor antagonists within 7 days or had unstable systemic diseases (e.g., cardiovascular, pulmonary, autoimmune) or severe allergic conditions were also excluded.

## Clinical evaluation

### i-ImmunDx™ platform

The i-ImmunDx™ Analyzer is a highly sensitive reader specifically designed for Seinda’s POCT strips. It features a fast, user-friendly interface, allowing operators to efficiently test patient samples and access stored results. Compact, lightweight, and portable, it’s ideal for various POCT settings, including outpatient clinics, clinical laboratories, and inspection facilities. The analyzer offers two testing modes, providing flexibility to adapt to different workflow demands. It also supports automated results for qualitative, semi-quantitative, and quantitative *in vitro* immunoassays, enhancing testing efficiency and accuracy.

### Standard scoring for traditional conjunctivitis

There were 12 observation indicators, including 6 symptoms (itching, tearing, burning sensation, foreign body sensation, blepharospasm, photophobia) and 6 signs (vision, conjunctival congestion, secretions, conjunctival papillae, limbal glenoid hyperplasia, corneal fluorescein staining) (Table 1). Symptoms were investigated using a questionnaire and signs were scored according to slit lamp microscope (BQ900IM900) and its photographic device (Haag-Streit, Switzerland). The other symptoms and signs were classified into none, mild, moderate, and severe, which were recorded as 0,1,2, and 3 points, respectively.

**Table 1:** Clinical grading of ocular surface signs of allergic conjunctivitis.

Sites	Signs	Grading	Description
Conjunctiva	Congestion	Mild	Small dilation of conjunctival blood vessels
		Moderate	Between mild and severe
		Severe	The conjunctival blood vessels are so dilated that they cannot be distinguished
	Edema	Mild	Regional conjunctival edema
		moderate	Diffuse mild edema of the whole conjunctiva that does not rise above the conjunctiva sac
		Severe	Diffuse mild edema of the whole conjunctiva, higher than the conjunctiva sac
	Nipples	Mild	Area involved < 1/3 upper eyelid conjunctiva area
		Moderate	The area involved is 1/3 to 1/2 of the upper eyelid conjunctiva area
		Severe	Area involved > 1/2 upper eyelid conjunctiva area
Limbus corneae	Horner-Trantas nodules	Mild	1 to 4 nodules
		Moderate	5 to 8 nodules
		Severe	> 8 nodules
Cornea	Corneal epithelial lesions	Mild	Punctate epithelial shedding
		Moderate	Flaky epithelium shedding
		Severe	Corneal shield ulcer

### Method for detecting total tear IgE

The tear fluid sample is required to use a microvolume liquid collector to collect the tear fluid sample, namely sampling. Acquisition process: (1) gently hold the back tube of the trace liquid collector with the thumb and index finger. Pay attention not to squeeze the back tube and do not block the small hole on the top of the rear tube. (2) Collect collection: ask the collector to tilt the head to the side of the collected eye, and the eyes look up so that the tears converge in the outer corner of the eye. The tip of the capillary head is placed in the tear river of the outer eye, and the tears will automatically enter the capillary head. (3) After the collection, ensure that the sample is filled with the tip of the head capillary. Drop the collected tears into an IgE detection card (Seinda Biomedical Corp.). Place the card analyzer and read the parameters (21).

The analytical validation of the i-ImmunDx™ tear IgE POCT device was performed using tear samples from 217 clinically diagnosed cases, including 110 patients with AC and 107 with non-AC. The cohort included both male (*n* = 63) and female (*n* = 154) subjects, with an age range from 8 to 84 years. Receiver operating characteristic (ROC) curve analysis was conducted using MedCalc statistical software. The optimal IgE cut-off value was determined to be 22.08 IU/mL, yielding a sensitivity of 82.73% and a specificity of 82.24%. This threshold suggests that total tear IgE concentrations ≤22.08 IU/mL are indicative of non-AC.

### Statistical analysis

All data was entered into an Excel spreadsheet and analyzed using SPSS statistical software version 25 (IBM, Chicago, Illinois). Continuous data is presented as mean ± SD for normally distributed data. As appropriate, patient-related observations were compared with either the Correlation, One-Way ANOVA, or chi-squared tests. After performing univariate logistic regression analysis to screen for the differential diagnosis of type-IV AC, multivariate logistic regression analysis (forward: LR) was used confirm the independent correlation. Medcalc version 22.007 analyzed the cut-off value of IgE, receiver operating characteristic (ROC) curve analysis and optimal intercept value of clinical indicators. All tests were two-tailed, and a *p*-value of <0.05 was considered statistically significant.

## Results

### Clinical profile

Based on diagnostic criteria and preoperative evaluation, the study enrolled 254 patients (182 male, 72 female) with AC diagnosed between June 01, 2023, and June 01, 2024. All patients presented with frequent eye redness and itching. Of these patients, 113 (44.48%) were non-IgE AC group, while the other 141 (55.52%) IgE AC group. The mean age of the patients was 14.11 ± 12.46 years. The demographic characteristics of the two groups showed notable differences. Although sex distribution was not significantly different between the Non-IgE and IgE groups (male: 68.42% vs. 74.28%, *p* = 0.064), the mean age was significantly lower in the IgE group (11.44 ± 8.98 years) than in the Non-IgE group (17.45 ± 15.15 years, *p* < 0.001). None of the patients had eye movement restrictions or diplopia. The average eye itch score, tearing, secretion, blink situation, and foreign body sensation did not differ significantly between the two groups (*p* > 0.05), the remaining details are described in Table 2. In addition to the categorical clinical symptoms and signs summarized in Table 2, we also analyzed three continuous or semi-quantitative parameters. The conjunctival papillae scores were significantly higher in the IgE group compared to the Non-IgE group (1.09 ± 0.77 vs. 0.76 ± 0.80, *p* = 0.001). Similarly, the total conjunctivitis scores were elevated in the IgE group (4.33 ± 1.49) compared to the Non-IgE group (3.79 ± 1.48, *p* = 0.005). No significant difference was observed in the congestion scores between groups (1.00 ± 0.20 vs. 0.96 ± 0.26, *p* = 0.233). These findings provide additional quantitative support for more severe ocular surface inflammation in the IgE group. The antihistamine treatment in the IgE group showed a significant effect, while the combination of topical cyclosporine and intense pulsed light therapy in the Non-IgE group demonstrated better long-term control.

**Table 2:** Frequency of positive clinical symptoms and signs in the non-IgE and IgE groups.

Clinical symptom or sign	Non-IgE group (*n* = 114)	IgE group (*n* = 140)	*p* value
Eye redness, *n* (%)	60 (52.63%)	90 (64.28%)	0.117^‡^
Eye itch, *n* (%)	79 (69.29%)	104 (74.28%)	0.055^‡^
Tearing, *n* (%)	5 (4.38%)	11 (7.85%)	0.070^‡^
Secretion, *n* (%)	24 (21.05%)	39 (27.85%)	0.078^‡^
Foreign body sensation, *n* (%)	28 (24.56%)	32 (22.85%)	0.019^‡^
Frequent blinking, *n* (%)	38 (33.33%)	39 (27.85%)	0.059^‡^
Dust mite allergy, *n* (%)	9 (7.89%)	21 (15.00%)	0.109^‡^
Allergic rhinitis, *n* (%)	44 (38.59%)	54 (38.57%)	<0.001^‡^

Only positive findings are shown; percentages are based on the total number of patients in each group.

^‡^chi-squared test.

### Clinical score and IgE findings

**Figure 2:**
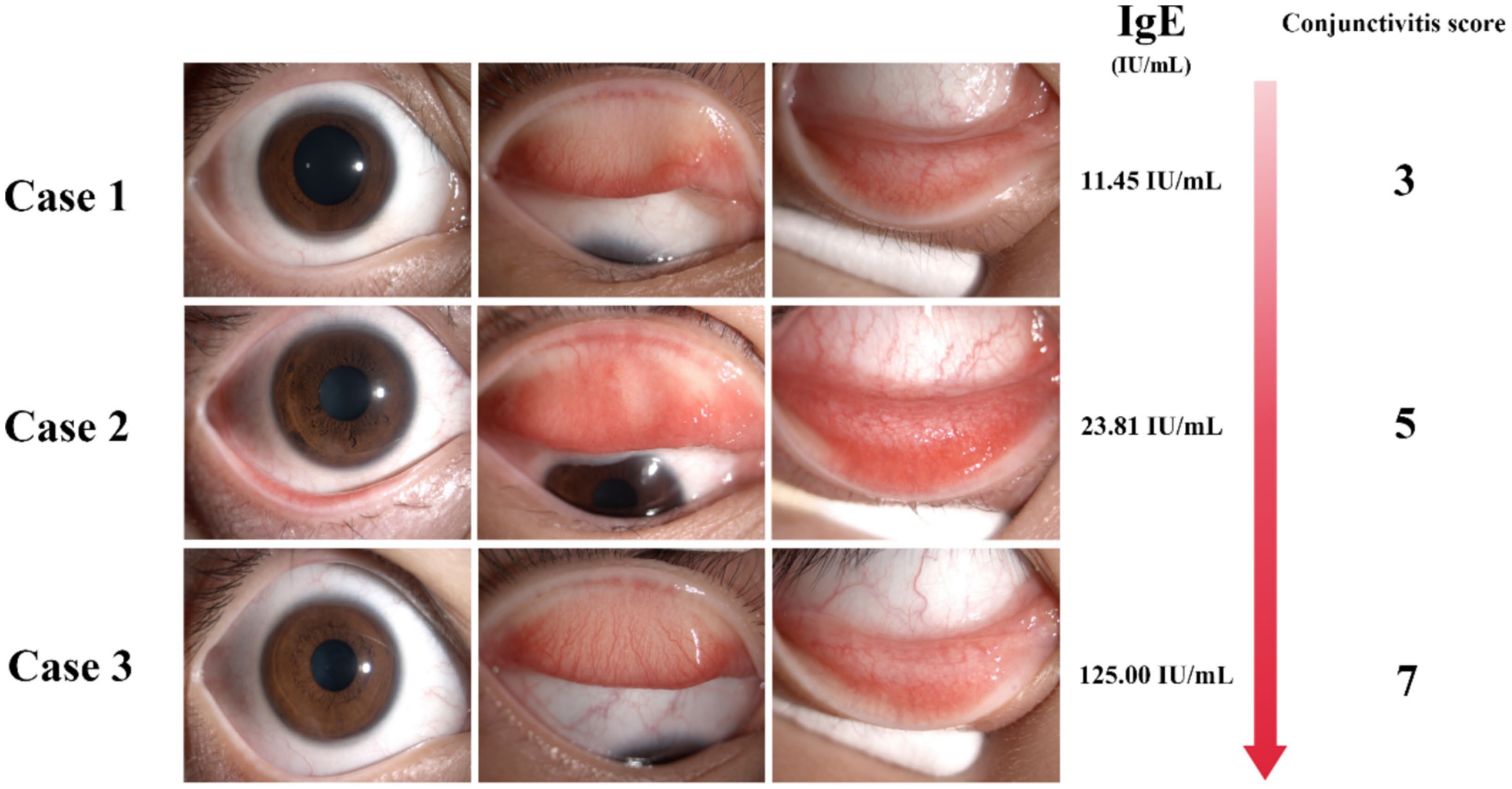
The IgE detection of tear POCT is correlated with the symptoms and signs of clinical patients. Detection of total tear IgE by POCT technology in patients with the presence of tear IgE with the increasing IgE concentration accompanied by the increase of conjunctivitis score.

The mean conjunctivitis score was 4.09 ± 1.51, and the mean tear IgE concentration was 7.73 ± 16.76 IU/mL. Tear IgE levels were <1 IU/mL in 44.1% of patients, 1–22.08 IU/mL in 45.2%, and >22.08 IU/mL in 10.7%. A negative correlation was observed between tear IgE concentration and age (*p* < 0.05), while positive correlations were found with conjunctival secretions, conjunctival papillary hyperplasia, and conjunctivitis scores (*p* < 0.05). No significant correlation was found between tear IgE levels and dust mite allergy or history of rhinitis. In cases where IgE can be detected, patients with conjunctivitis show stronger symptoms and signs in the clinic with increased IgE levels (Figure 2). The details are described in Table 3.

**Table 3:** Correlation test between IgE and clinical trials.

Variables	IgE	
	*R*	*p*
Sex	−0.103	*p* = 0.101
Age	−0.202	*p* = 0.001*
Congestion score	0.110	*p* = 0.080
Conjunctival papillae	0.133	*p* = 0.035*
Conjunctivitis scores	0.250	*p* < 0.001*
Eye redness	0.116	*p* = 0.066
Eye itch	0.084	*p* = 0.183
Tearing	0.055	*p* = 0.385
Secretion	0.133	*p* = 0.035*
Foreign body sensation	0.067	*p* = 0.286
Frequent blinking	−0.113	*p* = 0.072
Dust mite allergy	0.101	*p* = 0.107
Allergic rhinitis	0.060	*p* = 0.342

IgE, immunoglobulin E.

*p < 0.05.

### Univariate and multivariate analysis of differential factors in diagnosing type-IV allergic conjunctivitis

Type-IV AC was defined as the dependent variable (0 = type-I AC, 1 = type-IV AC) to analyze statistically significant factors associated with AC subtype differentiation. Univariate analysis revealed that age, total tear IgE, conjunctival papillae, were significantly associated with AC subtype (Table 4).

**Table 4:** Univariate and multivariate logistic regression analysis of vision does not improve.

Variables	Univariate analysis			Multivariate analysis		
	OR	OR (95%CI)	*p*	OR	OR (95%CI)	*p*
Sex	1.167	0.591–2.306	0.657			
Age	1.039	1.004–1.076	0.028*			
Congestion score	1.066	0.297–3.825	0.922			
IgE	0.840	0.797–0885	<0.001*	0.806	0.755–0.861	<0.001*
Conjunctival papillae	0.639	0.436–0.936	0.021*	0.009	0.108–0.724	0.009*
Conjunctivitis scores	0.918	0.753–1.119	0.398	3.113	1.750–5.537	<0.001*
Eye redness	0.989	0.537–1.824	0.973			
Eye itch	1.110	0.573–2.151	0.757			
Tearing	0.798	0.247–2.580	0.706			
Secretion	0.650	0.335–1.260	0.202			
Foreign body sensation	1.269	0.607–2.649	0.527			
Frequent blinking	1.925	0.933–3.975	0.076			
Dust mite allergy	0.587	0.252–1.368	0.217			
Allergic rhinitis	0.667	0.363–1.224	0.191			

*p < 0.05.

Multivariate logistic regression analysis (forward: LR) was conducted to further identify independent predictors. Model included general clinical factors such as tear IgE, conjunctival papillae and conjunctivitis scores reflecting clinical or point-of-care diagnostic data (Table 4).

To evaluate the diagnostic efficacy of the models, ROC curves were generated. The area under the ROC curve (AUC) for tear IgE alone was 0.896 (95% CI: 0.852–0.931), with an optimal cut-off value of 5.57 IU/mL. Tear IgE concentrations ≤5.57 IU/mL were indicative of non-type-IV AC, yielding a sensitivity of 89.00% and a specificity of 77.78%.

When tear IgE was combined with conjunctival papillae and total conjunctivitis scores to construct a multivariable diagnostic model, the AUC further increased to 0.912 (95% CI: 0.863–0.961), demonstrating superior discriminative performance. This combined model achieved a sensitivity of 81.50% and a specificity of 88.89%, indicating excellent diagnostic accuracy for distinguishing between type-I and type-IV AC.

## Discussion

The clinical course, duration, severity, and complications of AC vary, depending in part on the specific eye tissue affected and the local and systemic immune mechanisms (22). However, diagnosing this condition becomes particularly challenging in cases presenting mild and atypical symptoms, thus increasing the risk of misdiagnosis (23). The diagnostic approach toward AC is multifaceted, heavily relying on clinical evaluation complemented by laboratory assays. As revealed through this investigation, tear IgE levels are paramount in accurately mirroring the severity of AC. Hence, rapid tear IgE assays emerge as a crucial diagnostic tool by offering direct insights into ocular allergic reactions. In this study, we utilized the ability of POCT to rapidly deliver molecular-level information to provide a rapid understanding of molecular-level information in clinical IgE or non-IgE AC patients.

Previous research has shown that tear IgE levels correlate with total serum IgE levels (24). In patients with AC, total tear IgE levels are significantly higher than those in healthy individuals and are linked to the severity of various clinical signs of AC. This implies that tear IgE levels rise with increasing severity of AC (25). Bao J. reported that symptom improvement and therapeutic response in patients with SAC were associated with a decrease in tear IgE concentration. Moreover, patients who experienced recurrence after treatment exhibited significantly higher tear IgE levels than those without (26). Thus, measuring total tear IgE can serve as a valuable diagnostic tool for AC, accurately reflecting disease severity and prognosis.

In contrast to immunochromatography and enzyme-linked immunosorbent assays (ELISA), which are limited by their inability to precisely quantify tear IgE and are unsuitable for patients with insufficient tear production (25), the i-ImmunDx™ platform used in this study allows for the collection of minimal tear samples and the quantification of total IgE within 15 min. This method significantly enhances clinicians’ ability to differentiate AC in outpatient settings and make more informed treatment decisions. However, in this study, we observed that a subset of patients diagnosed with AC exhibited low tear IgE levels, which we attribute to Type IV hypersensitivity-driven AC.

In this study, we compared the symptoms of IgE-positive and IgE-negative patients, with the former showing more severe conjunctivitis, highlighting the value of integrating IgE tear testing in the clinical assessment of AC. Despite being the least abundant immunoglobulin in normal human serum, IgE plays a crucial role in allergic reactions (27, 28). In the eye, IgE binds to Fc receptors on mast cells, and upon allergen re-exposure, triggers the release of histamine, leading to itching and edema (29). Elevated tear IgE levels are associated with allergic seasons and household allergen exposure (30). Thus, measuring tear IgE not only aids in diagnosing AC but also reflects IgE involvement in local immune responses, potentially disrupting ocular surface homeostasis. Different subtypes of Type I AC have been shown to exhibit varying levels of tear IgE in previous studies (31). However, there are still limitations regarding the clinical utility of IgE testing in guiding treatment strategies (32).

Low tear IgE levels may indicate an autoimmune response due to loss of self-tolerance, contributing to the development of AC via a Type IV hypersensitivity mechanism (33). IV hypersensitivity AC represents a delayed hypersensitivity reaction at the conjunctival epithelium in response to endogenous microbial proteins or toxins. Management includes antihistamines, desensitization therapy, and symptomatic treatments (34). Treatment of IV hypersensitivity AC typically involves addressing underlying conditions, such as acute conjunctivitis or blepharitis, and improving overall health (35). The combination of topical corticosteroids and mast cell stabilizers has proven insufficient in effectively managing severe cases and preventing relapses (36). In contrast, non-steroidal immunomodulators such as Cyclosporine A and Tacrolimus have effectively treated type IV hypersensitivity reactions. These immunomodulators help regulate the immune response in more severe or chronic cases, offering better control where traditional therapies may fall short (37).

This study has several limitations. As a retrospective study, we were unable to ensure consistent follow-up for all patients, including those who declined follow-up despite effective treatment, which affected the completeness of outcome data and limited longitudinal analysis. Additionally, the absence of a healthy control group restricts the ability to define what constitutes elevated tear IgE levels. The single-center, single-population design reduces the generalizability of the findings, and the lack of an external validation cohort further limits the applicability of the results. Potential pre-analytical variability—such as prior use of artificial tears or anti-inflammatory agents—was not fully controlled and may have influenced tear IgE measurements. Although tear IgE levels showed significant correlation with conjunctivitis scores, the relatively weak strength of these correlations reduces their standalone clinical relevance. Elevated tear IgE may reflect disease severity but is unlikely to be the sole determinant of clinical presentation. Given the complex and multifactorial nature of AC, tear IgE levels should be interpreted within a broader diagnostic context rather than as an independent marker. Furthermore, the absence of long-term follow-up data prevents assessment of IgE dynamics over the course of treatment. Overall, the moderate-to-weak correlations between tear IgE and clinical parameters, combined with these design limitations, suggest that future studies should include healthy controls, adopt multicenter and prospective designs, control for pre-analytical variables, and incorporate structured follow-up to better validate tear IgE as a robust biomarker for AC.

**Figure 3:**
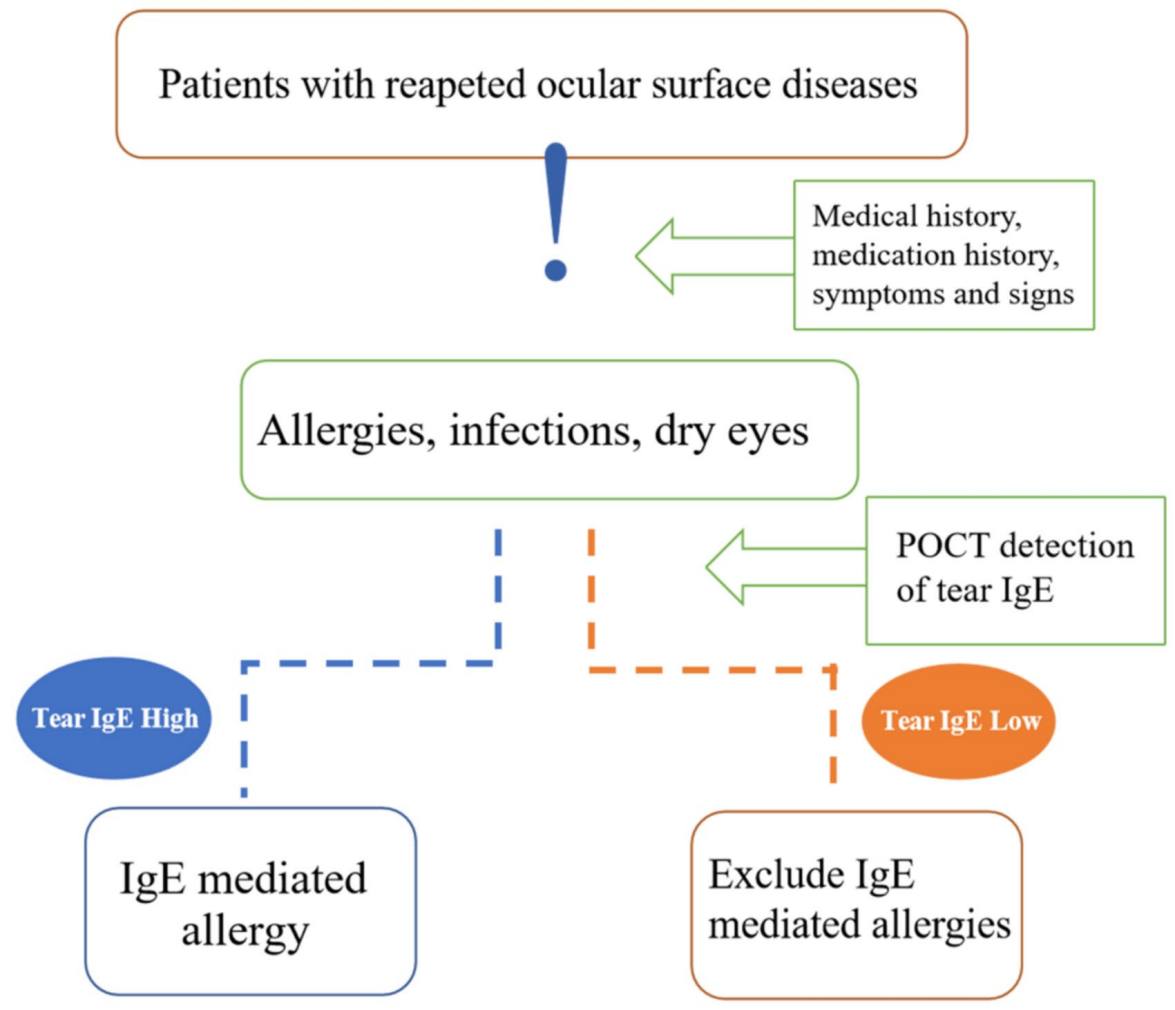
Diagnostic clinical value of tear IgE. Tear IgE can be a tool for differential diagnosis in patients with repeated visits for ocular surface diseases, such as infectious conjunctivitis and dry eyes that require a differential diagnosis with allergic conjunctivitis. Others are drug-toxic knots, membranitis, autoimmune keratoconjunctivitis, and lacrimal duct disease. We can know if inflammation is an IgE-mediated allergic reaction.

The reliability of tear IgE content as a biomarker is influenced by several pre-analytical variables, including ocular hygiene practices before sampling, using artificial tears, and administering anti-allergy medications (25, 30, 38). As a result, a comprehensive clinical evaluation, considering the specificities of each patient’s condition, is imperative in interpreting diagnostic tests. This investigation underscores the clinical utility of total IgE testing in elucidating the nature and progression of allergic reactions, facilitating the differential diagnosis of allergies through positive IgE findings, and informing management strategies tailored to the individual patient’s IgE profile (Figure 3). The findings advocate for the adoption of point-of-care tear IgE testing as a valuable adjunct in the diagnosis and longitudinal management of AC, bolstering its clinical utility.

## Conclusion

The quantitative detection of basic tear IgE provides an objective basis for the molecular level of hormone use in clinical allergic patients. The objective means of differential diagnosis for patients with atypical symptoms and abnormal transient appearance. The rapid detection of tear IgE offers a promising, safe, and potentially reproducible approach for quantitatively assessing tear IgE levels in ocular surface diseases. Preliminary data suggest that tear IgE testing may aid in disease differentiation and management; however, further studies are required to confirm its clinical utility and validate its diagnostic value.

## Data Availability

The raw data supporting the conclusions of this article will be made available by the authors, without undue reservation.
